# Job Insecurity and Intention to Quit: The Role of Psychological Distress and Resistance to Change in the UAE Hotel Industry

**DOI:** 10.3390/ijerph192013629

**Published:** 2022-10-20

**Authors:** Asier Baquero

**Affiliations:** Department of Business Studies, Westford University College, UCAM, Al Taawun St., Sharjah P.O. Box 32223, United Arab Emirates; abaquero@ucam.edu

**Keywords:** job insecurity, intention to quit, psychological distress, resistance to change, hospitality, hotel industry, United Arab Emirates, PLS-SEM

## Abstract

Hotel organizations today are in a state of constant change due to high competition, the emergence of pandemics, and cyclical economic crises. Hospitality employees are currently affected by job insecurity. The purpose of this research was to investigate the effect of job insecurity on intention to quit among hospitality workers, integrating the mediating effect of psychological distress and resistance to change and their mutual relationship. A total of 312 surveys were completed in four four- and five-star hotels in the UAE (Dubai and Sharjah). The SmartPLS 4 software was used to test the hypotheses in a mediation model with the bootstrapping method. The results showed that all of the direct links were positive and significant, and mediating relationships were confirmed. This study found that job insecurity predicts intention to quit through psychological distress and resistance to change acting as mediators, and these factors themselves also impact significantly on intention to quit. Resistance to change is impacted significantly by job insecurity and psychological distress, which suggests that a deeper approach to employees’ resistance to change should be taken, especially when conducting performance appraisals in the hotel industry, by searching for its roots and aiming to minimize employees’ intention to quit.

## 1. Introduction

The ongoing global economic instability has devastated the travel, entertainment, restaurant, hotel, and other hospitality and service industries, forcing them to relook at their operations. Like other industries, the hospitality industry relies heavily on employees to project the respective organizations’ service delivery approaches [1]. In their view, the authors decry the increasing turnover rates in today’s hospitality industry as being due to low employee satisfaction levels, affecting hospitality companies’ financial performances. The uncertainties have also escalated workers’ feelings of job insecurity due to the shifting economic situations, causing counterproductive work behaviors [2]. Since researchers are still unsure about global economic stabilization, a critical question entails regarding how the current prevalence of job insecurity (JI) in the hospitality industry may affect employees’ intention to quit (ITQ), psychological distress (PD), and resistance to change (RTC).

A key contributor to prolonged JI is the COVID-19 pandemic. According to the United Nations Industrial Development Organization (UNIDO) 2020 report, global projections estimate COVID-19-related job losses at 100.8 million and “a $2.7 trillion decrease in GDP in the travel and tourism sector,” making its impacts “five times worse than the 2008 financial crisis” ([3], p. 71). However, hospitality companies have implemented specific recovery measures to generate more profits and retain their workers. For instance, the Hotelier in the Middle East reported the second-highest hotel occupancy worldwide in 2020 in the period following the lifting of most restrictions [3]. Despite such positive attainments, [4] admitted that COVID-19 has negatively impacted workers’ attitudes, augmenting their turnover intentions. Reference [5] insisted that, like other global regions, the Middle East is experiencing a tourism recession, rendering workers in the service or hospitality industries jobless and uncertain of their future. The same extends to Dubai, with [3] indicating that workers still fear the pandemic’s long-term impacts and the required long-standing changes to achieve efficiency improvements. As a result, very few foresee their long-term retention in the industry, leading to high ITQ and the seeking of other, better opportunities. 

Despite contingency plans by hospitality businesses to grow past the pandemic, the generated sense of JI in employees has been pointed out, changing their intentions to remain and “sense of trust in their company” ([6], p. 1). Reference [7] concurred with these assertions, adding that the economic instability created by the COVID-19 aftermath has created unfavorable attitudinal constructs among hospitality workers, especially regarding their treatment in jobs and their working conditions. Since the latter is harmful, many workers have declined to accomplish the set mandates, with some citing the unlikeliness of staying in their respective hospitality or service organizations [7]. The Middle East and Dubai are no different, with their hospitality industries also experiencing unmotivated employees who are unlikely to commit to their job positions and roles. 

Reference [8] found that hospitality employees view change as an external threat that causes emotional distress, making them react negatively to it. Due to the high rates of JI within the hospitality sector, workers are constantly scared about any change initiative due to the perceived threat to their employment [8]. RTC is usually evaluated in hotel employees’ performance appraisals, and this is evaluated as something negative and intrinsic to the person, without taking into account that there may be other factors that are causing this state, and that this can impact their ITQ. Evidence suggests that change is a painful process due to the unpredictable situation that the employees find themselves in [9]. Similar findings were obtained by [10], who noted that emotional distress due to work-related stress could result in dissatisfaction, thus making employees want to quit.

The ongoing global inflation and several international military tensions have also established unfavorable grounds for hospitality industries to assure their workers of job security. Although there is limited research related to inflation’s relationship with JI in the hospitality industry, [11] asserted that today’s inflation, which has been escalated by the COVID-19 pandemic, high energy prices, and supply chain disruptions, has created a foggy future for workers, with many projecting JI. While researchers have not paid direct attention to these issues in the Middle East and Dubai’s hospitality industries as of yet, the above literature depicts the facilitation of JI, PD, and RTC, which can be expected to increase workers’ desire to quit. 

The hotel industry needs happy employees to achieve high customer satisfaction and loyalty [12]. However, this is one of the sectors that faces a lot of uncertainty due to the ever-changing business environment. The seasonality of tourism, the emergence of pandemics, and variation in economic conditions lead to unpredictability for hospitality workers. JI is presently a major concern for hotel employees, who remain uncertain about the future of their income. Insecurity regarding their job is a leading stressor and cause of PD, because it is a reaction to an external situation that one has no control over. An emotionally distressed worker will become defensive and adopt avoidance habits, resulting in resistance to any change initiative. At the same time, anxiety and stress due to JI can reach a level beyond one’s coping abilities, leading to the voluntary decision to quit.

The aim of this research was to propose a model that shows the impact that JI has on the ITQ, PD, and RTC of hotel workers, as well as the impact that PD has on RTC, and specifically the mediating effect of both PD and RTC between JI and ITQ, fulfilling the gap in the existing literature. In contrast to earlier studies, the suggested model has a novel feature that involves the mediation of both PD and RTC.

The main outcome of this research is the validation of the model, with the hypotheses assessed through PLS-SEM, where all the direct links were positive and significant, and where mediating relationships were confirmed. JI significantly impacts ITQ, PD, and RTC, with a higher influence on PD. PD significantly impacts RTC and ITQ, with a higher influence on ITQ. RTC also significantly impacts ITQ. Both PD and RTC significantly mediate the relationship between JI and ITQ.

**Figure 1:**
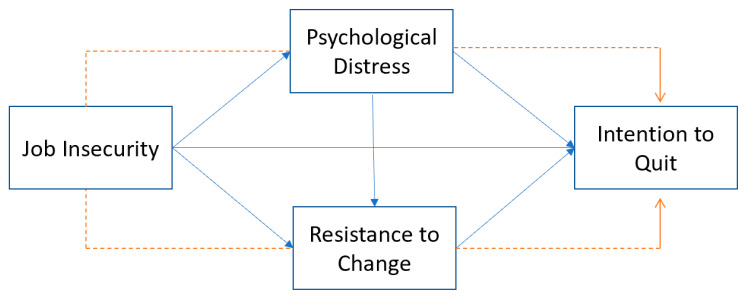
Conceptual framework.

PD might be considered a specific concept to be determined by a non-psychology professional, but RTC is a concept that is habitually used by hotel and human resources (HR) managers when evaluating their staff. This research proves that PD and RTC mediate the positive relationship between JI and ITQ. Additionally, RTC is impacted by PD and impacts itself on ITQ. Therefore, a new approach to RTC should be taken by hotel and HR managers when conducting hotel employees’ performance appraisals, searching for its roots and aiming to minimize employees’ ITQ. Figure 1 displays the study’s conceptual framework.

## 2. Literature Review and Hypothesis

### 2.1. Job Insecurity and Intention to Quit

JI refers to workers’ fear of becoming unemployed after losing their current jobs. JI is considered “a source of stress that damages employees’ psychological and physical health and reduces their motivation” ([13], p. 41). Reference [14] agreed with the claim that JI increases with work stress, which can push individuals beyond their capacity to cope. In return, work stress leads to a decline in employees’ psyches, with many opting to quit. JI occurs in two forms, namely, affective and cognitive JI, including anxiety about possible job loss and possible benefits from job loss, respectively [13]. The concept cuts across industries, with some researchers [13,15] viewing it as a vital contributor to workers’ display of lower efforts to attain set organizational goals due to reduced willingness to spend energy and time on work. The hospitality/service industry has had its workers facing JI due to the changing economic dynamics, which, unfortunately, has led to high turnover intentions. 

Reference [16] discussed on how employees’ intents and attitudes toward their jobs are affected by perceived job insecurity. On 942 workers in 3 distinct industries (food industry, retail, and education) in Spain, they investigated two hypotheses. First, it was determined that job insecurity has a negative impact on job satisfaction and organizational commitment and a favorable impact on intention to quit. Next, it was determined that job insecurity, economic need, and employability interact to predict these outcomes.

In a similar vein, the research by [17] revealed that organizational justice and the organizational justice climate both moderated the association between job insecurity and job satisfaction as well as the intention to quit the organization.

A critical contributor to JI and ITQ in the hospitality industry entails social loafing due to a limited supportive work environment. Social loafing occurs because of a “reduction in the amount of effort and motivation of individuals when working together compared to working individually” ([13], p. 42). Since the hospitality industry is highly involving, these researchers suggest that social loafing often lowers employees’ willingness to continue working in their work environments. Apart from limited group cohesion and heightened role stress, a key contributor to social loafing and employees’ ITQ involves a lack of support for workers. Reference [18] discussed social support, pointing out that since the service/hospitality industries thrive through favorable worker–customer interactions, employees require adequate social support to feel that they have job security. The researcher maintained that social support enhances the feelings of being loved, valued, and cared for by others. Adopting it in any hospitality organization reduces workers’ intentions to quit. Other related concepts involve support from top management and co-workers [13,19,20]. Researchers have indicated that it is unlikely for workers to experience JI and develop turnover intentions when they receive enough support from fellow employees and organizational leadership. Besides improved customer retention, adequate support lowers social loafing, thereby reducing employee turnover. 

According to [21], studying the nursing profession in Europe, nurses’ experiences of job insecurity can be lessened if they believe that the healthcare organization they work for values their input and cares about them.

One’s success in the hospitality industry depends on one’s employability. However, one’s attainment of job security extends to the willingness to offer needed services without sabotage [22]. Unfortunately, with the changing environment created by economic instability and the COVID-19 pandemic, hospitality sector employees have struggled to develop the required skills to enhance their job security. As a result, Ref. [22] pointed out an increase in service sabotage, with many employees feeling intense pressure to deliver the required services. This process has contributed to job stress, since employees consider their work environment unfavorable, pushing them to develop turnover intentions [14]. Therefore, based on this review, it is evident that JI in the hospitality/service industries and ITQ occur due to work-related stress, social loafing, and inadequate social, co-worker, and top management support.

**Hypothesis** **H1.** *Job insecurity has a significant relationship with intention to quit.*

### 2.2. Job Insecurity, Psychological Distress, and Resistance to Change

Recently, many organizations have been downsizing, with such restructuring considered a normal strategic human resource activity, yet it comes with adverse emotional disturbances to employees [23]. Reference [24] defined JI as a “subjective perception of feelings” that one’s job is not safe, creating a belief that they could soon lose their work. It arises when there is uncertainty about the future existence of the job [25]. A feeling of JI can lead to the development of stress. According to [23], stress can push one to participate in specific activities as a coping mechanism, resulting in severe emotional distress. A study [26] of Chinese firms also supports the results that the fear of job loss can lower employees’ self-esteem, leading to serious mental instability. This is more prevalent among newly employed permanent staff who are starting their careers and who rely on employment to meet their basic needs [26].

Reference [27] used a latent deprivation model to illustrate that perceived job loss is “stressful because it threatens the satisfaction of the fundamental needs fulfilled by employment,” including status and income. Empirical evidence supports these results, revealing that JI threatens mental health because of unpredictability, resulting in frustration in attempts to react accordingly because of uncertainty [27]. The COVID-19 pandemic has proven to be an extremely challenging time, exposing workers to fear related to job security when most firms shut down. According to [28], workers in the hotel industry have commonly been exposed to emotional distress due to the risk of job loss as restaurants and hotels close. According to the conservation of resources theory, people experiencing JI view situations as more threatening, resulting in lowered psychological wellbeing. The employees in the hotel industry have witnessed significant job loss during the pandemic, reducing their emotional and cognitive resources. A quantitative study by [29] also supports the findings that workers in the hospitality sector are the most likely to develop PD due to perceived JI. 

Previous studies on the role of JI in RTC have examined psychological contract violation as a model to determine the link. According to [30], the contract model perspective assumes that employees exchange their labor for wages; hence, JI is a violation of the contract between the company and workers. Change comes with a lot of uncertainties, thus making employees’ jobs unpredictable. Reference [31] agreed with other empirical evidence that when people perceive change as a threat to their job, they are more likely to resist. This is an emotional response to stressors over which they lack control, making them take a reactive attitude toward anything perceived as a threat. Similar results were found by [32], who revealed that employees withdraw emotionally and behaviorally in situations that create unpredictability in their jobs. They become less satisfied, demotivated, and uncommitted to every activity within the firm, leading to reactive response mechanisms to protect their wellbeing. RTC is, therefore, a counter-mechanism in which people reject certain initiatives they deem to be a threat to their work [33].

A meta-analysis and systematic review by [33] examined the role of job security on employees’ attitudes. Based on attitudinal theory, job attitude precedes work behaviors, including productivity and support to change initiatives. Reference [34] examined the effects of mergers within the hospitality industry and found that employees resist such initiatives because of the increased perceived job loss and reduced work engagement. More workers in this sector develop a negative attitude toward a change initiative when no clear communication is made, making them feel that their work is at stake [34]. Due to the role of employees in change management, perceived JI remains a key factor in the failure of most change initiatives [35]. Years of experience also play a critical role in the extent of PD in employees. However, limited studies have examined how JI impacts employees differently based on their years of work and experience, creating a gap in the literature.

**Hypothesis** **H2.** *Job insecurity has a significant relationship with psychological distress.*

**Hypothesis** **H3.** *Job insecurity has a significant relationship with resistance to change.*

### 2.3. Psychological Distress, Resistance to Change, and Intention to Quit

The success of any change initiative depends entirely on the attitude and reaction of the employees toward it. Reference [36] defined PD as a state of anxiety and depression that occurs when a person reacts to an emotional disturbance from the external environment over which they have limited control. This can include discomfort, fear of loss, and external threats to one’s stability. Change propels people to move from comfortable situations to discomfort due to the fear and uncertainty it creates within the organization [36]. A study by [37] also supports the assertion that the change process can evoke emotions, thus impacting the behavior of individuals, which finally determines how they respond to change. When workers undergo a cognitive response to the situation, their thinking and sense-making processes change, causing people to adopt actions that will protect their social capital and wellbeing. Reference [38] explained psychological capital as a positive emotional state of development that allows people to develop self-esteem. PD lowers mental stability, resulting in a response that alters behavior. This process makes them less ready for change, because it creates uncertainty, especially regarding their job security [38]. 

Change processes within an organization usually result in the disruption of daily operations, which causes much unpredictability. According to [39], change can erode the predictability of activities within the organization, leading to frustration and confusion. The ability of workers to manage change through resilience determines how they react to it. Resultantly, the extent to which one can manage stress and fear and develop psychological capital plays a crucial role in their attitude toward the change process [39]. On the contrary, [40] took a different approach by examining how PD impacts workers’ “threat appraisal and the subsequent withdrawal cognitions and behaviors.” However, this study found similar results, indicating that emotional distress increases the chances of developing a withdrawal attitude, and thus RTC [40]. 

Psychologists analyzing the mental process behind RTC have recognized the role of people’s “depressive paradox,” which makes them avoid change efforts. The fear of job loss creates psychological pain for employees, encouraging an avoidance state. The “investment model of resistance” assumes that mentally distressed persons will be more motivated to avoid further actions that can cause fear, rather than engaging in potentially beneficial activities [41]. The pain causes PD, which pushes people into a state of rejection because of further uncertainty. In this cognitive process, change is viewed as an unpleasant scenario, which creates an attitude of resistance [41].

Many researchers analyzing the impacts of PD on employees’ intention to leave use psychological contract theory. According to [42], a psychological contract involves a form of reciprocal agreement between the employer and the employee, with each party expected to meet their obligations. In a study conducted within the hospitality industry, workers were more likely to leave when they perceived unfairness and a psychological contract breach [42]. Reference [10] defined ITQ as a voluntary decision by workers to leave a company and seek work elsewhere when they no longer feel committed to their current workplace. Hospitality remains one of the most stressful sectors due to the nature of the job, such as the low job security because of the seasonality of the business. Stress emerges when an employee cannot control the difficult situations at work and resorts to a reactive mechanism as a survival technique [10]. Reference [43] also agreed with [10] that PD can create low commitment and job dissatisfaction, leading to an increased desire to quit. 

The effects of depression on turnover have mainly been studied in nurses during the COVID-19 period, as many of these workers have experienced frustration, trauma, and loss of control [44]. The traumatic events surrounding the pandemic have caused many to consider leaving the nursing profession. In a survey by [45], the findings indicated that distressed employees are four times more likely to consider leaving their workplace. The decision depends on the severity of the PD, with 55% of people experiencing extreme stress saying they will leave [45]. Much of the research in this area has used longitudinal and meta-analysis designs. Therefore, future studies must utilize experimental designs and randomized controlled trials to generalize the findings.

**Hypothesis** **H4.** *Psychological distress has a significant relationship with resistance to change.*

**Hypothesis** **H5.** *Psychological distress has a significant relationship with intention to quit.*

### 2.4. Resistance to Change and Intention to Quit 

In the current dynamic business environment, change has become an integral part of any company. However, introducing change is more likely to result in voluntary turnover, because employees view the process as a shock, owing to the many uncertainties involved [46]. According to [46], change creates new demands, which require workers to put in more effort to realize the new destination, thus creating stress and burnout. In response, workers are likely to voluntarily leave the job if they cannot deal with the pressure. Reference [47] found that the perceived change impact and one’s ability to cope could drive workers to resist change until a certain level beyond which they feel powerless and decide to leave. Change is usually accompanied by several reorganizations, downsizing, and restructuring, which is likely to create fear, forcing workers to use defense mechanisms. Similar findings were found by [48], who noted that employees only feel comfortable working in a company that they are committed to, with a disruption of commitment resulting in the desire to leave. 

Reference [49] viewed RTC as a rejection of a loss of a valuable thing by moving from the known to the unknown. The fear of the unknown and the threat of loss of economic fulfillment remain key factors that motivate people to resist change. Resisting change by employees only becomes problematic when it results in turnover due to the leadership’s failure to react early enough [50]. According to [50], managers can utilize RTC as an opportunity to successfully lead the organization when they communicate to counter the message of fear amongst workers. However, failure to act results in reduced commitment, PD, and ITQ. A study with hospitality industry workers also affirmed that change is an organizational stressor, which can mediate itself through counterproductive behaviors such as ITQ [43]. When pressure for change becomes unbearable, employees will experience levels beyond which they cannot cope further. To this extent, many will leave their work. 

One of the leading strategies to reduce turnover during the change management process is to manage resistance and alleviate fear. Using Lewin’s change model, [51] revealed that by communicating the change and bringing everyone on board, management can reduce the pain of change. The pain associated with change management remains a key factor that makes people feel that they lack control of the situation and results in them deciding to quit [52]. Building trust, being transparent, and effectively communicating the change can reduce pain and create comfort. The restructuring and downsizing aspects of change have been examined as the primary stressors because they lead to a sense of JI [53]. If they are unable to predict what the future holds for them within the company, workers may consider looking for alternatives early rather than wait for a problem to occur [54]. Sufficient evidence indicates that 70% of change initiatives fail because leaders fail to consider the emotional aspects of the employees, leading to withdrawal and desire to leave [55].

**Hypothesis** **H6.** *Resistance to change has a significant relationship with intention to quit.*

### 2.5. The Role of Psychological Distress and Resistance to Change

The mental wellbeing of employees has been studied broadly over recent decades due to the importance it has for the success of any organization. A study by [56] examined the concept of PD from the perspective of JI. This investigation showed that JI is the greatest psychological risk to employees in any workplace and can result in dissatisfaction, lack of commitment, withdrawal, and resistance to any change attempts [56]. Research into the hospitality sector also supports the findings that JI creates psychological strain and anxiety amongst frontline workers, making them unable to perform effectively [57]. Mental distress arises due to the fear of losing future income, which causes a reaction to a situation over which one has limited control [57]. Reference [58] also supported the discovery that JI is a chronic and prevalent organizational stressor and a leading factor in developing counterproductive behaviors. The existing literature identifies JI as a predictor of counterproductive behaviors within a workplace, because it inhibits the psychological contract between the employer and the employee, thus having a negative impact on health and wellbeing [58]. Counterproductive behaviors arise due to limited affective commitment, resulting in increased opposition to any change initiative and willingness to exit voluntarily [13]. 

Recent studies have also linked job stress to counterproductive behaviors such as aggression, hostility, low productivity, RTC, and ITQ [59]. The key job stressors, such as perceived JI and burnout, are known to cause dissatisfaction amongst workers. Studies, therefore, have found a strong link between PD and the intention of employees to voluntarily quit their jobs [59]. When the stress levels go beyond coping abilities, a person is most likely to consider themselves in a helpless situation, and with no control over the situation, they choose to leave.

Researchers in the area of turnover within the hospitality sector have argued that the concepts of JI, PD, RTC, and ITQ are intercorrelated [14]. In a study on the connection between these terms, [60] found that JI causes emotional instability, making workers adopt defensive measures by avoiding change. When the pain of the change process becomes unbearable and one cannot cope further, they choose to leave. While under distress, employees will adopt counterproductive behaviors, such as remaining silent and mentally withdrawing from all organizational activities, a process that leads to the decision to resign [61]. At this point of resistance, a firm requires a charismatic leader who can engage and communicate with the staff to alleviate the fear of change before it becomes unmanageable [62]. Companies that poorly manage change are more likely to record the highest levels of employee turnover.

**Hypothesis** **H7.** *Psychological distress mediates the relationship between job insecurity and intention to quit.*

**Hypothesis** **H8.** *Resistance to change mediates the relationship between job insecurity and intention to quit.*

## 3. Materials and Methods

### 3.1. Participants and Procedure

Data were collected between 1 and 31 July 2022 from four hotels located in the UAE, specifically in Dubai and Sharjah. The hotels were rated as four- and five-star establishments, some being purely city business hotels, while others were purely vacation resorts. All four hotels were managed by an international hotel chain. The Regional Area Manager was contacted, the research purposes were explained, including anonymity for employees and the hotel chain, and authorization was obtained.

A total of 400 questionnaires were distributed by a designated person, liaising with the HR manager of each hotel. Through the aforementioned Regional Area Manager, communication with HR management and approval were attained. The questionnaire’s respondents received no compensation. Of these, 312 questionnaires were returned as valid samples, providing a response rate of 78%. The sample employees worked directly for the hotel, for an outsourced company offering temporary services (casual staff in housekeeping, restaurants, etc.), or for an external company running a business in the hotel (SPAs, various outlets, outsourced restaurants and bars, and entertainment).

The survey consisted of 40 Likert 1–5-scale items (see Appendix A) and five sociodemographic profile questions (see Table 1). The items assessed JI, ITQ, PD, and RTC. A team of specialists composed of academics from Spanish and UAE universities (3) and experts in hospitality (3) approved the questionnaire. The experts looked for grammatical faults as well as how the questions’ original context and intended audience would be interpreted by responders. Academics were assisted by hospitality professionals in understanding the reality of the make-up of the hotel staff in the UAE, and hotel professionals were assisted by university academics in understanding the value of rigorous methodology when developing a questionnaire using constructs from earlier studies. Minor text changes were suggested by the panel, which also recommended keeping the original number of entries.

The suggested model and accompanying hypothesis tests were assessed using partial least squares (PLS), which was satisfactory for the number of respondents (N = 312). 

### 3.2. Survey Instruments

JI was measured through a 10-item scale adapted from [63]. A sample item is, “One cannot feel secure in a job at any given point of time of their career.” This scale’s Cronbach alpha (reliability measure) was 0.87. ITQ was measured through a 10-item scale adapted from [64], and a sample item is, “There is an excessive workload and time pressure at my workplace.” The reliability for this scale was 0.84. PD was measured through a 10-item scale adapted from [65], and a sample item is, “After COVID-19, I stay away from others as much as possible.” The Cronbach’s alpha of this scale was 0.87. RTC was measured through a 10-item scale adapted from [66], and a sample item is, “I would rather be bored than surprised.” The Cronbach’s alpha was 0.92.

### 3.3. Common Method Variance

A highly important issue in a survey sample is common method bias. This study examined the common method bias using Harman’s single-factor test [67]. From [68], the single-factor test was developed to ascertain if CMV existed among the constructs. According to the data, all sample items could be broken down into 40 different factors, with the first factor accounting for less than the stated threshold of 50%, or 34.405%, of the total variance. Additionally, we used SmartPLS to carry out a complete collinearity assessment test. Reference [69] and several other social science scholars have claimed that this is a method that is comparatively accurate and effective [70,71]. All VIF values were substantially below the suggested cutoff of 5, indicating that this model does not suffer from the usual process bias [69].

Table 1 shows the demographic statistics of the sampled individuals in this research.

## 4. Results

The SEM approach has been extensively utilized due to its potential to explain unique regression associations in a unified framework and test. Therefore, it is feasible to use this approach to determine interaction/mediation effects. The significance of PLS-SEM for both forms of studies (confirmatory and exploratory) was the primary reason behind the selection of this approach [72]. According to [72], SEM can be categorized into two types, PLS-SEM and covariance-based SEM (CB-SEM). CB-SEM is primarily employed to approve/reject theories, while PLS-SEM supports expanding and advancing theoretical knowledge [73]. PLS-SEM analysis approaches are carried out in two phases: measurement model assessment and structural model estimation [72]. PLS-SEM is appropriate for complicated and multi-order constructs and is equally helpful for limited sample sizes [73]. PLS-SEM calculates path coefficients and factor loadings during the statistical analysis process to reduce parameter estimation biases [74]. For this study, SmartPLS 4 was employed to analyze the data. Most recent management research projects have used the PLS-SEM method for data analysis [75,76,77].

### 4.1. Measurement Model

**Figure 2:**
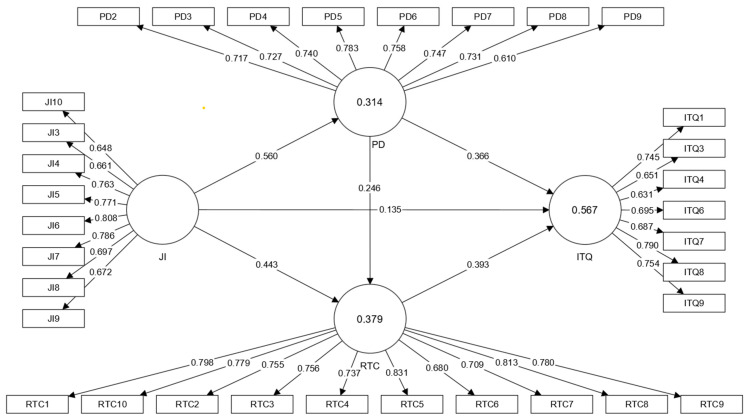
Measurement model.

Table 2 and Figure 2 demonstrate the measurement model results for the latent constructs, which show that the outer loading of each indicator was greater than 0.60 for all constructs and satisfied the rule of thumb [72]. Items JI1, JI2, PD1, PD10, ITQ2, ITQ5, and ITQ10 were rejected due to lack of loadings. Furthermore, all the AVE values ranged from 0.503 to 0.585 for the reflective constructs, supplying proof of the measurements’ convergent validity. Every detected indicator strongly influenced its corresponding latent variable (i.e., JI, ITQ, PD, and RTC). Each latent variable adequately explained more than 50% of its indicator variance. Moreover, the outcomes of the reflective measurement models showed that the instrument has high internal consistency because JI (0.900), ITQ (0.876), PD (0.900), and RTC (0.934) have relatively high CR values (above the suggested threshold of CR > 0.7). Similarly, the Cronbach’s alpha scores of ITQ (0.835), JI (0.872), PD (0.873), and RTC (0.921) were above 0.70. Thus, the findings show that the reflected measurement models satisfy the necessary evaluation standards. In addition, the variance inflation factor (VIF) and T-statistics results also support the measurement model results and confirm the model robustness.

The discriminant validity of the present study framework was assessed in three ways. First, the Fornell–Larcker criterion was used to assess the discriminant validity of the reflective constructs. According to the criteria established by [78], if the top (first) value of each column is the maximum after taking the square root of the AVE of each element, then it implies the establishment of discriminant validity [78]. As shown in Table 3, discriminant validity based on the Fornell–Larcker criteria was confirmed, since the top value of the variable associations in each column was the maximum for all constructs. Second, the heterotrait–monotrait ratio of correlations (HTMT) technique was employed because it better determines discriminant validity between constructs. The HTMT approach looks at the proportion of correlations between two constructs and at the correlations within them. According to the HTMT ratios criteria, the HTMT ratios’ values must be <0.85, although values up to 0.90 are appropriate [72]. As seen in Table 3, all HTMT ratios were <0.85, suggesting that the current research model’s discriminant validity is confirmed. 

Next, the cross-loadings were also used to measure the discriminant validity, and the results indicated that all data complied with the criteria. All indicators loaded as high (>0.6) on their respective variables but low on others. The difference between the item score with its parent construct and those of the item to the other variables was greater than 0.1, meeting the suggested criteria. This also confirms the discriminant validity of the model. The cross-loading results for the latent constructs are presented in Table 4. 

The *F*^2^, *R*^2^, and *Q*^2^ were also estimated to evaluate the model’s robustness (see Table 5). To calculate how much a predicting (exogenous) variable contributes to an endogenous variable’s *R*^2^ value, effect sizes (*F*^2^) were determined. The findings in Table 5 show that the study’s variables had impact sizes that ranged from medium to high, supporting the model’s robustness [72]. Next, the *R*^2^ and *Q*^2^ values for ITQ (*R*^2^ = 0.567; *Q*^2^ = 0.267), PD (*R*^2^ = 0.314; *Q*^2^ = 0.162), and RTC (*R*^2^ = 0.379; *Q*^2^ = 0.210) were determined, which supported the model’s sample predictive power [79], and the model’s predictive relevance in terms of out-of-a sample prediction was confirmed by the results of blindfolding with an omission distance of seven, which showed *Q*^2^ values well above zero [72].

### 4.2. Structural Model Assessment

**Figure 3:**
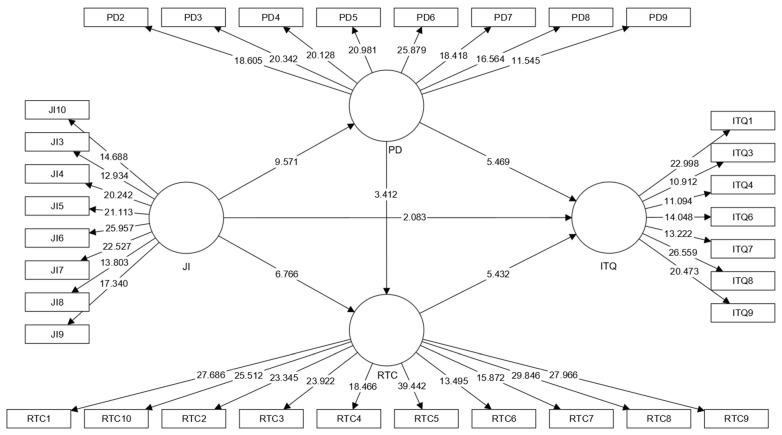
Structural model.

After critical evaluation of the measurement model, the structural model tests were evaluated in the second phase. The bootstrap resampling technique with 5000 resamples [80] was used to demonstrate the importance of both direct and indirect approaches. Table 6 and Figure 3 demonstrate the hypothesis results of the direct and indirect associations.

First, the direct relationships were assessed before evaluating the mediation effects. The results in Table 6 reveal that JI significantly impacts ITQ, PD, and RTC. Specifically, JI’s influence on PD (β = 0.560, *p* < 0.001) was more significant than its effect on ITQ (β = 0.135, *p* < 0.037) and RTC (β = 0.443, *p* < 0.001). Thus, the findings confirm that H1, H2, and H3 were supported. 

For H4 and H5, this study proposed positive effects of PD on RTC and ITQ. The findings confirmed that PD has a significant impact on both variables, but its influence on ITQ (β = 0.366, *p* < 0.001) was comparatively greater than on RTC (β = 0.246, *p* < 0.001). Therefore, H4 and H5 were both supported. 

For H6, Table 6 demonstrates the positive influence of RTC on ITQ (β = 0.393, *p* < 0.001), and the findings support H6. 

To test the mediation effect, the bootstrapping indirect effect method [81] was used with a 5000 resample. For H7 and H8, this study proposed a mediating effect of PD and RTC in the relationship between JI and ITQ. The results revealed that PD (β = 0.205, *p* = 0.001) and RTC (β = 0.174, *p* < 0.001) significantly mediate the relationship between JI and ITQ. Thus, H7 and H8 are supported. 

Lastly, the PLS predict technique was employed, with 10 ten-fold cross-validation and 10 replications, to evaluate the out-of-sample predictive ability of the model. The PLS-SEM RMSE numbers were then compared to those from a simplistic linear benchmark (RMSE of the linear model (LM)) in the PLS to predict the output. According to a general rule for prediction models [82], more predictive ability is shown by lower values for all PLS-SEM RMSE (or MAE) measurement indicators compared to all those of the LM RMSE. While lower values for the majority of the PLS-SEM RMSE measurement indicators compared to those of the LM RMSE correspond to medium predictive power, lower values for the minority of the PLS-SEM RMSE measurement indicators compared to those of the LM RMSE relate to minor predictive capacity. Additionally, lower values for the model’s predictive capability are shown by greater values for all of the measurement indicators of the LM RMSE compared to those of the PLS-SEM RMSE (or the MAE). The results validated the medium predictive capacity of the model by comparing the reduced prediction errors of the PLS-SEM RMSE analysis relative to the naïve benchmark shown in the LM RMSE output (see Table 7).

## 5. Discussion and Conclusions

The purpose of this research was to deepen the study of human resources management (HRM) concepts affecting hotel employees today (JI, ITQ, PD, and RTC), and to propose a model that shows their relationship and allows us to understand their impact and managerial implications.

This research empirically studied the effect of JI on ITQ among hotel employees, integrating the mediating effect of PD and RTC and the mutual relationship between PD and RTC. This was assessed through 312 surveys completed in four four- and five-star hotels in the UAE in July 2022. This research used the SmartPLS 4 software package to test hypotheses in a mediation model with the bootstrapping method.

The findings can be summarized as follows:

First, the proposed model was validated, since all of the direct links were positive and significant, and mediating relationships were confirmed. 

Second, JI was found to significantly impact ITQ, PD, and RTC, with a greater influence on PD. A meta-analysis by [83] summarized 13 studies between 1997 and 2013 on job security and found that while employment offers mental stability, employed people are most likely to develop stress due to fear of work loss. This study utilized a large sample size, thus increasing the credibility of the outcome.

Third, PD was found to significantly impact RTC and ITQ, with a greater influence on ITQ. A study by [84] examined the impacts of emotional distress on the decision to leave the organization from the perspective of social and economic exchanges. While social exchange entails the relationship between the employer and the employee, an economic exchange involves materialistic benefits such as income and rewards. Any situation that threatens an employee’s income, including salary cuts and the possibility of job loss, is likely to create mental disturbances [84]. At this stage, the worker will try to cope, but only to a certain level, beyond which one becomes powerless and decides to quit [84].

Fourth, it was found RTC also impacts significantly on ITQ. A quantitative study [85] on the intention of nurses to leave recognized that voluntary resignation begins as a withdrawal process when one is unable to cope with external threats, such as change. Even though they will try to resist, these processes are beyond their control, leaving them powerless. When there is limited organizational support during the change initiative, one feels threatened and may opt to seek an alternative job [86]. 

Fifth, it was found that both PD and RTC significantly mediate the relationship between JI and ITQ. A study [87] of a Japanese factory discovered that job security strongly correlates with employee retention. The results of this investigation indicated that dissatisfaction and lack of commitment at work are a product of insecurity amongst staff, leading to higher turnover [87]. The turnover rate is, therefore, a consequence of PD and fear that emerges when employees feel that their jobs are not secure [14].

Although hotel occupancy in the UAE has increased in recent years, COVID-19 has had a detrimental influence on employee sentiments and increased turnover intentions. Similar to other global regions, the Middle East is going through a tourism crisis, which has left unemployed and insecure individuals in the service and hospitality sectors, who continue to worry about the pandemic’s long-term effects. Because so few people predict their long-term retention in the sector, their ITQ is high and they look for other, better opportunities [3,4,5].

Accordingly, the implications of the results at all levels (hotel business management, worker and customer satisfaction, and human resources) are discussed next. 

The main managerial implication from this research for organizations is that a more thoughtful approach should be taken toward employees’ state of PD and RTC, especially in times when it is obvious that JI is affecting employees, so ITQ can be minimized.

Both internal and outsourced employees’ job satisfaction is affected by the way managers lead their teams, due to the special factor of human relationships in hospitality [88]. Many other factors can also affect hospitality employees’ job satisfaction. Employees exhibit undesirable behaviors when they feel intimidated by the possibility of losing their current jobs in the future. This is a significant problem in the hotel business because most staff have direct contact with clients, and clients can easily collect on an employee’s emotions and find themselves less satisfied.

PD might be a specific concept to be determined by a non-psychology professional, but RTC is a concept that is habitually used by hotel and HR managers when conducting performance appraisals. This research proved that RTC mediates the positive relationship between JI and ITQ. RTC is impacted by both JI and PD, and impacts itself on ITQ. Therefore, a new approach toward RTC should be taken by hotel and HR managers when conducting performance appraisals in order to minimize employees’ ITQ.

Despite its contributions, this research has some limitations that future research could address. First, the four hotels in the sample were located in the same country, the UAE, and managed by the same hotel company. Future studies could analyze hotels in different countries and hotels managed by different hotel companies. Second, questionnaires were collected during the month of July 2022 for this cross-sectional survey. Collecting questionnaires in various phases over time, as a longitudinal study, could add value to this research. Third, the model analyzed the mediation effect of PD and RTC. Further understanding of this topic could be gained by including analyses of the moderating influences, such as gender and others, and by incorporating control variables, such as the type of work contract or the length of current employment, when appropriate.

## Figures and Tables

**Table 1:** Demographics.

Category	Sub-Category	Frequency	Percent
Gender			
	Male	172	55.1
	Female	140	44.9
	Total	312	100.0
Age			
	18–25 years old	38	12.2
	26–35 years old	116	37.2
	36–45 years old	107	34.3
	>45 years old	51	16.3
	Total	312	100.0
Position			
	Team member	158	50.6
	Team leader	123	39.4
	Management	31	9.9
	Total	312	100.0
Experience			
	Less than a year	55	17.6
	1–3 years	84	26.9
	4–6 years	90	28.8
	>6 years	83	26.6
	Total	312	100.0
Contractual relationship			
	Permanent	172	55.1
	Outsourced	140	44.9
	Total	312	100.0

**Table 2:** Measurement model results.

Constructs	Items	Loadings	VIF	T-Statistics	CA	CR	AVE
Intention to Quit	ITQ1	0.745	1.571	22.998	0.835	0.876	0.503
	ITQ3	0.651	2.033	10.912			
	ITQ4	0.631	2.019	11.094			
	ITQ6	0.695	1.692	14.048			
	ITQ7	0.687	1.724	13.222			
	ITQ8	0.790	2.694	26.559			
	ITQ9	0.754	2.241	20.473			
Job Insecurity					0.872	0.900	0.531
	JI3	0.661	1.470	14.688			
	JI4	0.763	1.581	12.934			
	JI5	0.771	1.934	20.242			
	JI6	0.808	2.195	21.113			
	JI7	0.786	2.350	25.957			
	JI8	0.697	1.957	22.527			
	JI9	0.672	1.628	13.803			
	JI10	0.648	1.477	17.340			
Psychological Distress					0.873	0.900	0.530
	PD2	0.717	1.680	18.605			
	PD3	0.727	1.727	20.342			
	PD4	0.740	1.761	20.128			
	PD5	0.783	2.238	20.981			
	PD6	0.758	1.825	25.879			
	PD7	0.747	1.959	18.418			
	PD8	0.731	1.920	16.564			
	PD9	0.610	1.356	11.545			
Resistance to Change					0.921	0.934	0.585
	RTC1	0.798	2.483	27.686			
	RTC2	0.755	2.339	23.345			
	RTC3	0.756	1.991	23.922			
	RTC4	0.737	2.006	18.466			
	RTC5	0.831	3.019	39.442			
	RTC6	0.680	1.838	13.495			
	RTC7	0.709	2.204	15.872			
	RTC8	0.813	2.585	29.846			
	RTC9	0.780	2.232	27.966			
	RTC10	0.779	2.132	25.512			

Note: VIF, variance inflation factors; CA, Cronbach’s alpha; CR, composite reliability; AVE, average variance extracted.

**Table 3:** Discriminant validity.

	Heterotrait–Monotrait Ratio				Fornell–Larcker Criterion			
Constructs	ITQ	JI	PD	RTC	ITQ	JI	PD	RTC
ITQ					0.710			
JI	0.651				0.569	0.728		
PD	0.733	0.634			0.636	0.560	0.728	
RTC	0.711	0.632	0.534		0.653	0.581	0.494	0.765

**Table 4:** Cross-loadings.

Items	ITQ	JI	PD	RTC
ITQ1	0.745	0.473	0.515	0.670
ITQ3	0.651	0.364	0.540	0.353
ITQ4	0.631	0.374	0.480	0.392
ITQ6	0.695	0.417	0.445	0.357
ITQ7	0.687	0.287	0.347	0.340
ITQ8	0.790	0.454	0.416	0.541
ITQ9	0.754	0.408	0.384	0.485
JI3	0.331	0.661	0.314	0.334
JI4	0.420	0.763	0.393	0.438
JI5	0.406	0.771	0.360	0.482
JI6	0.489	0.808	0.416	0.452
JI7	0.463	0.786	0.443	0.481
JI8	0.369	0.697	0.334	0.364
JI9	0.441	0.672	0.475	0.397
JI10	0.363	0.648	0.492	0.407
PD2	0.487	0.405	0.717	0.357
PD3	0.455	0.368	0.727	0.394
PD4	0.470	0.395	0.740	0.405
PD5	0.425	0.421	0.783	0.319
PD6	0.460	0.482	0.758	0.487
PD7	0.374	0.413	0.747	0.306
PD8	0.422	0.400	0.731	0.255
PD9	0.580	0.359	0.610	0.310
RTC1	0.553	0.535	0.401	0.798
RTC2	0.499	0.566	0.397	0.755
RTC3	0.495	0.502	0.399	0.756
RTC4	0.412	0.405	0.345	0.737
RTC5	0.539	0.447	0.366	0.831
RTC6	0.342	0.295	0.207	0.680
RTC7	0.467	0.284	0.318	0.709
RTC8	0.504	0.414	0.417	0.813
RTC9	0.535	0.460	0.442	0.780
RTC10	0.582	0.448	0.421	0.779

**Table 5:** Effect size, coefficient of determination, and blindfolding results.

		*F* ^2^		*R* ^2^	*Q* ^2^
	ITQ	PD	RTC	Endogenous Constructs	Endogenous Constructs
ITQ				0.567	0.267
JI	0.024	0.457	0.217		
PD	0.199		0.067	0.314	0.162
RTC	0.222			0.379	0.210

**Table 6:** Hypothesis results.

Hypotheses	Relationships					Beta	STDEV	BCI-LL, BCI-UL		T Statistics	*p*-Values	Results
	IV		M		DV							
Direct Effects												
H1	JI	→			ITQ	0.135	0.065	0.011, 0.258		2.083	0.037	Supported
H2	JI	→			PD	0.560	0.059	0.440, 0.670		9.571	0.000	Supported
H3	JI	→			RTC	0.443	0.066	0.314, 0.569		6.766	0.000	Supported
H4	PD	→			RTC	0.246	0.072	0.105, 0.386		3.412	0.001	Supported
H5	PD	→			ITQ	0.366	0.067	0.236, 0.500		5.469	0.000	Supported
H6	RTC	→			ITQ	0.393	0.072	0.251, 0.531		5.432	0.000	Supported
Mediating Effects												
H7	JI	→	PD	→	ITQ	0.205	0.047	0.120, 0.304		4.405	0.000	Supported
H8	JI	→	RTC	→	ITQ	0.174	0.043	0.096, 0.264		4.101	0.000	Supported

Note: JI, job insecurity; PD, psychological distress; RTC, resistance to change; ITQ, intention to quit.

**Table 7:** PLS-Predict.

	PLS		LM		PLS-LM		*Q*^2^ Predict
	RMSE	MAE	RMSE	MAE	RMSE	MAE	
ITQ1	0.964	0.713	0.987	0.730	−0.023	−0.017	0.211
ITQ3	0.838	0.639	0.847	0.646	−0.009	−0.007	0.119
ITQ4	0.868	0.643	0.853	0.618	0.015	0.025	0.129
ITQ6	0.739	0.597	0.756	0.611	−0.016	−0.014	0.164
ITQ7	0.848	0.649	0.857	0.653	−0.009	−0.004	0.060
ITQ8	0.878	0.664	0.876	0.647	0.002	0.017	0.196
ITQ9	0.871	0.666	0.885	0.669	−0.014	−0.003	0.154

## Data Availability

The data presented in this study are available on request from the corresponding author.

## Appendix A

**Table A1:** Survey instrument.

Code	Questions
Job Insecurity (1 = Strongly Disagree to 5 = Strongly Agree)	
JI1	Job insecurity often results from the HRM practices of management.
JI2	Job insecurity causes lots of stress and poor wellbeing.
JI3	The sense of job insecurity affects the productivity of an employee.
JI4	Autocratic behavior of the manager or supervisor continuously instigates the feeling of losing one’s job at any time.
JI5	The continual changes in work practices post-COVID-19 often result in low adaptability, thus leading to the fear of losing one’s job.
JI6	Job insecurity often leads to the continuous thought of leaving a job and switching to a new one.
JI7	Better communication can mitigate the sense of job insecurity in employees.
JI8	The factor of job insecurity can be addressed by participative decision making in the company.
JI9	Increasing workers’ employability through adequate training can reduce job insecurity.
JI10	One cannot feel secure in a job at any given point of time in their career.
Intention to Quit (1 = Strongly Disagree to 5 = Strongly Agree)	
ITQ1	There is an excessive workload and time pressure at my workplace.
ITQ2	There is too much interference and disturbances in the work at the job.
ITQ3	Procrastination in work distresses me a lot.
ITQ4	There are no prospects for growth and promotion at work.
ITQ5	There is poor job security in my company post-COVID-19.
ITQ6	I get paid less than I work for my company due to COVID-19.
ITQ7	My job is affecting my mental health.
ITQ8	There are no or poor training and development prospects in my company.
ITQ9	I experience stagnation in growth by working in my current job due to the lack of opportunities post-COVID-19.
ITQ10	I keep on finding reasons to quit my current job.
Psychological Distress (1 = Strongly Disagree to 5 = Strongly Agree)	
PD1	I have the impression that I have messed up my life.
PD2	After COVID-19, I stay away from others as much as possible.
PD3	I have difficulties facing my problems.
PD4	Lately I have no patience.
PD5	After COVID-19, I am aggressive about everything and nothing.
PD6	I feel ill at ease with myself.
PD7	I feel stressed and under pressure.
PD8	I feel like throwing everything to the wind, quitting.
PD9	I am now less receptive to the ideas and opinions of others.
PD10	I have difficulty concentrating on anything post-COVID-19.
Resistance to Change (1 = Strongly Disagree to 5 = Strongly Agree)	
RTC1	I would rather be bored than surprised.
RTC2	I will take a routine day over a day full of unexpected events any time.
RTC3	I generally consider changes to be a negative thing.
RTC4	If I were to be informed that there is going to be a significant change regarding the way things are done at work, I would probably feel stressed.
RTC5	When I am informed of a change of plans, I tense up a bit.
RTC6	When things do not go according to plan, it stresses me out.
RTC7	If my boss changed the criteria for evaluating employees, it would probably make me feel uncomfortable, even if I thought I would do just as well without having to do any extra work.
RTC8	Changing plans seems like a real hassle to me.
RTC9	Once I have made plans, I am not likely to change them.
RTC10	I do not change my mind easily.
